# Childhood Threat and Deprivation and Links to Mental Health Behaviors and Health Risk Behaviors Among Young Sexual Minority Men: The Differential Roles of Mindfulness and Emotion Regulation

**DOI:** 10.3390/ijerph23050609

**Published:** 2026-05-05

**Authors:** Jennifer A. Poon, David G. Zelaya, Vedhalakshmi Rajasankar, Matthew J. Murphy, Riley A. Russell, Shufang Sun

**Affiliations:** 1 Department of Psychology, University of Alaska Anchorage, Anchorage, AK 99508, USA; japoon@alaska.edu (J.A.P.); rarussell2@alaska.edu (R.A.R.); 2 Department of Behavioral and Social Sciences, Brown University School of Public Health, Providence, RI 02903, USAvedhalakshmi_rajasankar@brown.edu (V.R.); matthew_j_murphy@brown.edu (M.J.M.)

**Keywords:** child abuse, child deprivation, sexual minority men, emotion regulation, mindfulness

## Abstract

Background: Young adult sexual minority men (SMM) disproportionately experience childhood interpersonal trauma. The Dimensional Model of Adversity and Psychopathology (DMAP) framework proposes that exposure to threat (i.e., emotional, physical, and sexual abuse) and deprivation (e.g., physical and emotional neglect) are differentially linked to adult psychopathology. Studies of predominantly heterosexual samples have revealed emotion regulation and mindfulness as mechanisms linking childhood trauma to mental health and health risk behaviors in adulthood. However, the influence of emotion regulation (ER) or mindfulness as associated with exposure to threat vs. deprivation has not been examined among SMM in adulthood. Objective: This study explored the relationships between childhood threat/deprivation and mental health and health risk behaviors among SMM with ER and mindfulness as mediators. Participants and Setting: The sample consisted of 317 SMM (*M*_age_ = 26.70; *SD* = 3.87; ages 18–35; 59.3% White) recruited from the community. Methods: Childhood experiences of threat and deprivation were assessed via the Childhood Trauma Questionnaire; ER difficulties and dispositional mindfulness were assessed using self-report. Mental health behaviors were assessed using a composite score consisting of self-reported depressive, anxiety, PTSD symptoms, and suicidality. Health risk behavior score included the Sexual Compulsivity Scale, Alcohol Use Disorders Identification Test, and smoking history (Y/N). Results: Threat and deprivation were both positively correlated with mental health and health risk behaviors. *Threat* was associated with both mental health and health risk behaviors via emotion regulation (ER) difficulties. *Deprivation* was associated with these outcomes through both ER difficulties and mindfulness. Conclusions: Treatment aimed at bolstering ER and mindfulness skills among those with histories of abuse and deprivation, respectively, may help reduce psychopathology risk among SMM.

## 1. Introduction

Sexual minority (SM) populations experience significant mental health inequities compared to their heterosexual counterparts [1,2,3]. Notably, emerging/young adulthood (ages 18–35) can be a stressful period that is associated with higher risks of psychopathology regardless of sexual orientation [4]; these risks are even more heightened for sexual minorities (e.g., behavioral risk factors, STI transmission, and psychopathology). Young adult sexual minority men (SMM) in particular experience higher rates of mental health behaviors, such as mood disorders, anxiety, and post-traumatic stress disorder (PTSD), in addition to health risk behaviors such as substance use and suicidality [5]. Meanwhile, the research field of sexual minority health has increasingly recognized childhood maltreatment as a contributor to vulnerability to these symptoms during young adulthood [6]. Childhood maltreatment—including physical, sexual, psychological, and emotional abuse and/or neglect—is a major public health concern [7]. The majority of reported cases to the U.S. Department of Health and Human Services (>3.9 million cases in 2021) involve multiple forms of maltreatment, including high rates of deprivation (76%), physical abuse (16%), and sexual abuse (10.1%). Meta-analyses have shown that, compared to heterosexuals, SM youth are more likely to experience more frequent, severe, and longer, sustained periods of maltreatment [8]. A retrospective survey of over 3000 respondents found that SMM reported significantly higher rates of childhood emotional and major physical maltreatment by both parental figures compared to their heterosexual counterparts [9]. Epidemiological studies reveal that the prevalence of childhood sexual abuse is 2–3 times higher for SMM compared to heterosexual men [10].

A primary limitation of the extant literature is the examination of childhood maltreatment as a unitary construct, precluding a more complete etiological understanding of how different forms of childhood adversity (e.g., abuse and deprivation) may uniquely confer risk for psychopathology. A fuller, more nuanced elucidation of how early interpersonal trauma may contribute to risk for mental health vs. health risk behaviors could inform prevention, early identification, and intervention efforts among populations at disproportionate risk of maltreatment, including young adult SMM. Accordingly, the innovative Dimensional Model of Adversity and Psychopathology (DMAP) framework has reconceptualized and, consequently, reclassified experiences of childhood adversity as primarily falling along one of two underlying dimensions, threat vs. deprivation [11], each of which may have distinct associations with neurodevelopmental processes and socioemotional functioning [12,13]. Exposure to threat includes experiences involving harm or threat of harm to the child, ranging from community violence to experiences of physical, sexual, and/or emotional abuse. In contrast, deprivation refers to the *absence* of expected inputs from the environment (e.g., cognitive and/or social stimulation) during the child’s development, including experiences of physical and/or emotional neglect.

Of note, minority stress—or experiences that occur due to being in a discriminative and non-accepting environment such as discrimination, microaggressions, and internalized homophobia—is an important driver of health disparities for sexual minorities [14,15,16]. Such forces of stress and oppression begin early in life, which may in part contribute to the high prevalence of childhood trauma among sexual minorities. However, despite recent research showing its high prevalence among sexual minorities, research and knowledge on identity and non-identity-based forms of childhood adversity, including maltreatment, among SMM have been lacking.

### 1.1. Childhood Threat and Deprivation: Links to Adult Psychopathology

The majority of youth in the US will experience at least one traumatic event by the time they reach adulthood [17]. This is particularly true among sexual minority youth; for example, one meta-analysis found that sexual minority youth were, on average, 2.9 times more likely to report childhood sexual abuse compared to their heterosexual counterparts [18]. Research has consistently documented that childhood exposure to threat in the form of physical, sexual, and/or emotional abuse is a salient longitudinal risk factor for virtually all types of psychopathology [19]. For instance, child threat exposure (i.e., abuse) has been causally linked to a range of mental health behaviors, including depression, anxiety, and post-traumatic stress disorder [20], as well as suicidal ideation and actual attempts [21]. With regard to health risk behaviors, childhood abuse has also been longitudinally associated with substance use, smoking, and risky sexual behaviors [20].

Links between deprivation (i.e., physical and/or emotional neglect) in childhood and adult psychopathology remain less clear, with mixed findings emerging across studies depending on the age and composition of the sample, as well as the methodological nature of the study (e.g., cross-sectional, longitudinal, and cohort). For example, one cross-sectional study found that, compared to abused children, deprived children were more likely to display mental health behaviors but less likely to exhibit health risk behaviors [22]. On the other hand, one adult-focused study of 199 men found significant associations between childhood deprivation and health risk behaviors [23]. Finally, Busso and colleagues found that deprivation was associated with general psychopathology, whereas threat was uniquely associated with health risk behaviors in adolescents [24].

In contrast to the emerging literature on links between threat and suicidality, the scant data on links between child neglect and adult suicide risk has yielded mixed findings. One study found that deprivation was *not* associated with increased odds of suicide ideation and attempt [25], whereas another study found that childhood emotional neglect was associated with current suicidal ideation in a college student sample [26]. Relevant to the present study, none of the aforementioned studies included data on participants’ sexual orientation. Overall, research with predominantly heterosexual samples shows robust associations between *both* threat and deprivation and symptoms of psychopathology [27,28,29], with particularly strong findings with regard to threat. However, despite recent conceptual and empirical work proposing that threat and deprivation may differentially impact adult psychopathology by influencing the brain and biological systems in unique ways [11,13], the literature has lagged behind in its identification of specific psychological mechanisms (e.g., emotion regulation and mindfulness) by which childhood threat and deprivation may confer risk for adult psychopathology. These gaps in the literature are especially apparent among minoritized populations, including young adult SMM.

### 1.2. Childhood Threat and Deprivation: Links to Emotion Regulation

One mechanism through which threat-related adverse experiences in childhood may confer risk for subsequent mental health and health risk behaviors is through its negative impact on the neurobiological processes underlying children’s developing emotion regulation abilities [30]. Directly implicated in all common forms of psychopathology [31], emotion regulation (ER) constitutes a promising, transdiagnostic mechanism that may help us better understand links between childhood threat and adult psychopathology. Adaptive ER skills have been linked to positive adjustment among youth and adults alike, including favorable health, psychological, social, and occupational outcomes [31,32,33]. Conversely, ER difficulties have been conceptualized and empirically validated as a transdiagnostic factor underlying many forms of psychopathology [34,35]. ER is particularly important to examine among young adult SMM, given its prominent role in Hatzenbuehler’s mediation framework of minority stress and validated by empirical studies linking its role to mental health concerns in this population [36,37,38].

Threat exposure has been associated with deficits in both automatic (i.e., involuntary) as well as conscious ER abilities [39,40] as well as poorer behavioral inhibition [41], all of which have been directly tied to symptomatology. Relevant to the current study, prior work has documented that ER problems partially explain the link between childhood abuse and the subsequent psychopathology [42]. Notably, ER difficulties are also strongly associated with suicidality [42]. A recent 18-month longitudinal study found that limited access to ER strategies emerged as an intervening variable in the link between childhood threat exposure and subsequent levels of suicidal ideation in a high-risk adolescent sample [43]. Relatedly, there has been a wealth of research documenting links between threat, ER, and psychiatric comorbidities (see [44] for a review). For example, Cloitre found that women with a history of abuse evidenced emotional dysregulation and interpersonal problems, which, together, had just as much of an adverse influence on the women’s functional impairment as their PTSD symptoms [45], suggesting ER is a promising, clinically relevant construct of interest.

In contrast to the extensive research on threat, research on links between deprivation and ER is quite limited. Research on absent or neglectful caregivers who fail to provide the necessary interactions needed for children’s optimal development results in deficits across domains of language [46], social competence [47], and emotion regulation skills [48,49]. Paralleling the aforementioned links between threat/abuse and ER, one study found that childhood deprivation (i.e., neglect) predicts emotional dysregulation, which, in turn, was related to health risk behaviors [50]. Notably, children who experienced deprivation did not differ from non-deprived youth in covert aggression, but had significantly higher levels of *overt* aggression [51], which is strongly related to difficulties in ER. Another study conducted by the authors who originally conceived of the DMAP framework revealed that poverty—a proxy for deprivation—predicted poor inhibition in an emotional context and poorer cognitive control—but not poorer ER [40]. Of note, this study did not assess or report the adolescent participants’ sexual orientation; to our knowledge, no study has investigated childhood neglect, ER, and symptoms of psychopathology among adults—much less young adult SMM. A relatively recent meta-analysis revealed that childhood maltreatment was negatively associated with emotion regulation [32]; however, the authors were constrained by the quality and measurement of prior studies, which overwhelmingly treated maltreatment as a unitary construct, precluding our ability to discern any potential differences between threat (i.e., abuse) and deprivation (i.e., neglect). Taken together, the specific links between *deprivation*, ER, and symptomatology remain unclear.

### 1.3. Childhood Threat and Deprivation: Links to Mindfulness

As defined by Jon Kabat-Zinn, mindfulness means paying attention, in the present moment, non-judgmentally [52]. Although mindfulness and ER are both related to self-regulatory capacities, they are distinct constructs, with unique neurobiological, psychological, and clinical correlates and consequences [53]. In particular, mindfulness refers to attention and awareness of the present-moment experience (i.e., how much one notices what’s happening in the moment), while emotion regulation is a multi-dimensional competency on managing one’s emotions, such as to accept, tolerate, and flexibly respond to different emotional states [53]. In contrast to *state* mindfulness, *dispositional* or *trait* mindfulness refers to an individual’s level of mindfulness in their day-to-day life [53,54]. In general, trait mindfulness is associated with reduced psychological distress [55,56] and can be conceptualized as a protective factor that may buffer against the development of psychopathology [56]. Accordingly, those who are innately lower in dispositional mindfulness are more susceptible to both mental health and health risk behaviors [57]. A systematic review and meta-analysis of over 100 studies, most of which were correlational, found that trait mindfulness was inversely associated with mental health behaviors and health risk behaviors [58]. Indeed, research on non-clinical adult samples found inverse associations between trait mindfulness and symptoms of depression [59,60,61,62], anxiety [63], PTSD [64], and Borderline Personality Disorder [65]—including suicidality. With regard to health risk behaviors, another meta-analysis found that mindfulness was positively associated with multiple health behaviors (e.g., physical activity and sleep) and negatively related to alcohol use (*r * =  −0.06) [66]; this is consistent with prior research documenting the inverse association between trait mindfulness and substance use [67]. Although no studies have directly examined links between trait mindfulness and sexual compulsivity, one large study of over 700 young adults found that mindfulness was negatively associated with the perpetration of physical and sexual dating violence [68].

Paralleling the broader literature on childhood maltreatment and adult psychopathology, research has shown that adults who have experienced threat and/or deprivation early in life (i.e., before the age of 18) demonstrate lower levels of trait mindfulness in adulthood [69]. A major weakness of the existing research is the lack of differentiation between threat and deprivation. These limitations notwithstanding, one study of 978 college students found that those who had experienced threat in childhood (i.e., sexual abuse, peer abuse, or sibling maltreatment) showed lower levels of dispositional mindfulness [70]. Few studies have directly examined childhood deprivation and trait mindfulness in adulthood. However, perhaps because deprived youth fail to receive the expected inputs necessary for healthy development, childhood deprivation has been specifically linked to deficits in executive functioning [71]—a multi-faceted construct implicitly related to one’s level of dispositional mindfulness. Indeed, trait mindfulness is positively associated with higher executive attention, working memory, and inhibitory control [72,73]—all of which constitute core executive functioning processes. In a systematic review and meta-analysis of 91 studies (*N* = 31,188), compared to threat, early-life experiences of deprivation had a stronger association with the executive functioning domains of inhibitory control and working memory [74], both of which are directly implicated in trait mindfulness. Therefore, it is highly plausible that trait mindfulness may be a significant indirect effect in the relationship between childhood deprivation and adult psychopathology. Though the research on links between threat and mindfulness is limited, given the promising findings for mindfulness-based interventions for survivors of child abuse [75], it is possible that early-life threat may also contribute to deficits in trait mindfulness and subsequent risk for psychopathology.

## 2. Current Study

It is necessary to differentiate between different sources and dimensions of adversity (e.g., threat and deprivation) and examine transdiagnostic mechanisms like ER and mindfulness in order to better elucidate specific mechanisms underlying mental health and health risk behaviors. This is particularly important to investigate among young adult SMM, given their risks of elevated mental health symptoms as well as childhood trauma. The current study examines whether ER difficulties and mindfulness may similarly or differentially account for the relationship between childhood threat vs. deprivation and symptoms of psychopathology among a community sample of young adult SMM.

While cross-sectional in nature, this study is the first of its kind to preliminarily examine the unique associations between exposure to childhood threat and deprivation, respectively, and the development of mental health behaviors and/or health risk behaviors. Examining potentially malleable, transdiagnostic mechanisms such as ER and mindfulness is key to developing and tailoring effective prevention and intervention strategies for sexual minority boys and men alike. Specifically, it is possible that ER difficulties would indirectly affect the relation between childhood threat (i.e., abuse) and both mental health and health risk behaviors among young adult SMM. Given the mixed literature, we did not make any specific predictions with respect to deprivation and ER. It could also be that mindfulness would indirectly affect the relation between childhood deprivation (i.e., neglect) and both mental health and health risk behaviors; due to the lack of prior research in this area, we did not make any specific predictions with respect to threat and mindfulness.

## 3. Method

### 3.1. Procedure

Data was collected through an online survey of gay, bisexual, and queer young adult men from June 2021 to May 2022. The current study is a secondary data analysis part of a larger study that examined minority stressors and HIV/STI risk among young SMM [76]. The survey was hosted on a secure Qualtrics XM platform (Qualtrics, Provo, UT, USA). The survey was advertised on social media platforms (e.g., Facebook and Instagram) and email listservs of LGBTQ+ organizations across the United States. Interested individuals completed a brief screening questionnaire. A rigorous process of data validation was conducted, including both machine and manual checks (detailed protocol in [77]).

Participants were eligible for this study if they were: (a) between the ages of 18 and 35; (b) fluent in English; (c) residing in the U.S.; (d) assigned male sex at birth; (e) HIV negative or unknown status; and (f) had ever had sex with a man. Participants were excluded if they reported (a) being assigned female sex at birth; (b) being transgender; (c) never having had sex with a man; or (d) living with HIV. Participants provided informed consent at both the screener and survey stages by choosing “I agree.” Informed consent was obtained from all participants involved in this study. Upon survey completion and verification (described by [77]), survey participants were sent a $15 Amazon gift card to compensate for their efforts. This study was approved by the Institutional Review Board at Brown University (Protocol #: 2004002698, 13 August 2021).

### 3.2. Measures

#### 3.2.1. Demographic Covariates

We included annual household income, race (0 = People of Color, 1 = White), and education level as covariates of childhood maltreatment. Annual household income was measured on an ordinal scale from 1 (Less than $10,000) to 7 (Over $100,000). Similarly, education level was measured on an ordinal scale from 1 (Less than HS) to 5 (Graduate school). We included these items as covariates, given their prior associations and influence on childhood maltreatment [78,79,80].

#### 3.2.2. Childhood Trauma Questionnaire

The Childhood Trauma Questionnaire (CTQ) [81] is a 28-item, self-report measure of maltreatment experiences in childhood. This scale has been validated among sexual minorities [82,83]. Participants rated 28 items on a five-point Likert scale (1 = never true, 5 = very often true).

*Childhood Exposure to Threat*. Fifteen CTQ items corresponding to experiences of abuse (i.e., sexual, emotional, and physical abuse subscales; e.g., “*People in my family hit me so hard that it left me with bruises or marks*.”) were summed to create a total score ranging from 15 to 75. The CTQ threat measure demonstrated high internal consistency in this sample (*α* = 0.94).

*Childhood Exposure to Deprivation*. Ten CTQ items corresponding to experiences of deprivation (i.e., emotional and physical neglect subscales; e.g., “*I believe I was emotionally abused; I believe that I was physically abused*.”) were summed to create a total score ranging from 10 to 50. The CTQ deprivation measure demonstrated good internal consistency in this sample (*α* = 0.81).

#### 3.2.3. Mental Health Behavior (Composite Score)

Mental Health Behaviors were measured as a composite score of self-reported anxiety (GAD-7), depression (PHQ-9), suicide risk (SBQ-R), and post-traumatic stress symptoms (PCLC). This methodological approach of computing a composite score that represents mental health behaviors’ composite score has been widely used in prior research of psychopathology that focuses on its internalizing/externalizing dimensions [84,85,86]. This composite score was created from the sum of four measure scores, each described below.

*GAD7: Generalized Anxiety Disorder—7 Item Scale.* The seven-item Generalized Anxiety Disorder-7 (GAD-7) [87] was used to assess anxiety symptom frequency in the past two weeks. Participants rated the seven items on a four-point Likert scale (0 = not at all, 3 = every day). Items were summed such that high totals indicated greater anxiety symptom frequency. Specifically, scores in the 0–4 range are considered minimal anxiety, the 5–9 score range suggests mild anxiety, scores in the 10–14 range suggest moderate anxiety, and scores greater than 15 suggest severe anxiety. The GAD-7 demonstrated good internal consistency in this sample (*α* = 0.87).

*PHQ9: Patient Health Questionnaire—9 Item Scale.* The nine-item Patient Health Questionnaire depression module (PHQ-9) [88] of the PRIME-MD Diagnostic Instrument [89] was used to assess depression symptom frequency in the past two weeks. Participants rated the nine items on a four-point Likert scale (0 = not at all, 3 = nearly every day). Item responses were summed such that higher totals indicated greater symptom frequency. Scores in the 0–4 range suggest none or minimal depression severity, scores ranging in the 5–9 range suggest mild depression severity, scores ranging between 10 and 14 suggest moderate depression severity, scores ranging in the 15–19 range suggest moderately severe depression severity, and scores in the range of 20–27 suggest severe depression severity. The PHQ-9 demonstrated good internal consistency in this sample (*α* = 0.87) and in other samples of young adult sexual minority individuals [90,91].

*SBQ-R: Suicide Behaviors Questionnaire—Revised Edition.* The four-item Suicide Behaviors Questionnaire—Revised (SBQR) [92] assessed four dimensions of suicidality: lifetime suicide ideation and/or attempt, frequency of suicidal ideation over the past 12 months, threat of suicide attempt, and likelihood of suicidal behavior in the future. Individual item scores were summed such that higher totals indicated a greater self-reported suicide risk. The SBQR demonstrated good internal consistency in this sample (*α* = 0.84) and in other samples of young adult sexual minority individuals [93].

*PCLC: PTSD Checklist—Civilian Version.* The 17-item PTSD checklist assessed one’s agreement with a list of problems and complaints that individuals sometimes have in response to stressful life experiences (e.g., “*Repeated, disturbing memories, thoughts, or images of a stressful experience from the past*”) [94]. Participants rated the 17 items according to how much they had been bothered by each problem in the past month on a five-point Likert scale (1 = not at all, 5 = extremely). Item responses were summed such that higher totals indicated a greater post-traumatic stress severity. The PCLC demonstrated good internal consistency in this sample (*α* = 0.93) and in other non-clinical samples [94].

#### 3.2.4. Health Risk Behaviors (Composite Score)

Health risk behaviors were measured as a composite sum score of self-reported sexual compulsivity, hazardous and harmful alcohol consumption, and cigarette smoking history. This composite score was created from the sum of three measure scores, each described below.

*SCS: Sexual Compulsivity Scale.* The 10-item Sexual Compulsivity Scale [95,96] measured participant alignment with a list of problems and complaints that individuals sometimes have in response to sexual compulsions (e.g., “*My desires to have sex have disrupted my daily life*.”). Participants rated the 10 items according to how much they have been bothered by each problem on a four-point Likert scale (0 = not at all, 5 = very much). Item responses were summed to create a total score that ranged from 0 to 30. Higher totals indicated a greater overall sexual compulsivity. The SCS demonstrated good internal consistency in this sample (*α* = 0.89) and in other samples of SMM [97,98].

*AUDIT: Alcohol Use Disorders Identification Test.* The three-item Alcohol Use Disorders Identification Test (AUDIT-C) [99,100] measured consumption of alcoholic beverages in the past six months. Participants rated the frequency of their alcohol consumption (0 = never, 4 = four or more times per week), number of drinks consumed per day (1 = 1–2 drinks per day, 4 = 10 or more drinks per day), and how often they consumed more than six alcoholic drinks in one day (1 = never, 4 = daily or almost daily). Item responses were summed to create a total score ranging from 0 to 12, such that higher total scores reflect greater hazardous alcohol use in the past six months.

*Lifetime prevalence of cigarette smoking.* Lifetime prevalence of cigarette smoking was measured by the response (0 = no, 1 = yes) to the following question: “*Have you ever smoked cigarettes*?” [101].

#### 3.2.5. Difficulties with Emotion Regulation Scale

The Difficulties in Emotion Regulation Scale (DERS) [102] is an 18-item, self-report measure of emotion regulation issues. Participants rated the 18 items on a five-point Likert scale (1 = almost never, 6 = almost always). Example items include: “*I have difficulty making sense out of my* feelings,” “*When I’m upset, I become out of control, and* “*When I’m upset, it takes me a long time to feel better*.” Item responses were summed such that higher average scores indicated higher levels of emotion regulation difficulties. The DERS has been validated in prior research on sexual minorities [97] and also demonstrated good internal consistency in this sample (α = 0.90).

#### 3.2.6. Mindful Attention Awareness Scale

The 15-item Mindful Attention Awareness Scale (MAAS) [103] measured characteristics of dispositional mindfulness during one’s daily experience, which has also been validated and used among sexual minorities [104]. MAAS was selected since it measures dispositional mindfulness, capturing an individual’s trait or tendency to be at a state of mindfulness in one’s daily life. Participants rated the 15 items on a six-point Likert scale (1 = almost always, 6 = almost never). Example items include “*I find it difficult to stay focused on what’s happening in the present*,” “*It seems I am ‘running on automatic,’ without much awareness of what I’m doing*,” and “*I find myself preoccupied with the future or the past*.” Item responses were averaged such that higher average scores indicated higher levels of dispositional mindfulness. The MAAS has shown similar predictive validity with other mindfulness scales to well-being outcomes [105]. The MAAS demonstrated excellent internal consistency in this sample (α = 0.92).

### 3.3. Data Analysis

All data analysis was performed using R (version 4.4.1; R Core Team, Austria) in RStudio, a programming language and software environment for statistical computing [106,107]. The following packages were used for data cleaning and analysis: *dplyr* [108], *psych* [109], and *lavaan* [110]. Descriptive statistics and pairwise correlations were calculated between all variables of interest. For the associations between both dimensions of childhood trauma (i.e., threat and deprivation) and both dimensions of psychopathology (i.e., mental health behaviors and health risk behaviors), suggesting the existence of direct effects, we performed eight simple mediation analyses using bootstrapped regression-based path analysis. To do so, we constructed two models of mental health behaviors and two models of health risk behaviors, with each model treating either emotion regulation difficulties or dispositional mindfulness as the single mediator. Childhood experiences of threat and deprivation were treated as independent variables in each model, controlling for the covariates of age, race, education, and the countermeasure of childhood maltreatment (i.e., threat controlling for deprivation, and deprivation controlling for threat). We performed 1000 bootstrap samples to generate a 95% bias-corrected confidence interval of the indirect effect *a* × *b* [111]. Variables of interest were first transformed onto the z-distribution. In each mediation analysis, the *a* path represented the path from childhood maltreatment type to psychological mediators of emotion regulation or mindfulness, and the *b* path represented the effect of these psychological mediators on mental health behaviors or health risk behaviors in young adulthood. The output from our model also included path *c*, the total impact of the childhood maltreatment type on mental health symptoms in young adulthood, and *c*′, the direct impact of the childhood maltreatment type on mental health symptoms in young adulthood when accounting for the indirect effects of the psychological mediators. Variance inflation factors (VIF) were estimated to examine multicollinearity for the mediation models. Additionally, Bonferroni-corrected threshold was applied as a sensitivity analysis due to the presence of multiple comparisons. The significance threshold was set at *p* < 0.05 for all statistical tests.

## 4. Results

### 4.1. Participants Demographics

Following Mahalanobis’ distance test [112], multivariate outliers were identified and removed from the final analytic sample, which resulted in 317 young adults, all of whom identified as cisgender males and whose ages ranged from 18 to 35 (*M* = 26.70, *SD* = 3.87). Most participants identified as gay (80.8%, *n* = 256), followed by bisexual (18.0%, *n* = 57), and pansexual (1.3%, *n* = 4). Additionally, most participants identified their race as White (59.3%, *n* = 188), followed by Black/African American (35.6%, *n* = 113), multiple races (1.3%, *n* = 4), American Native or Alaskan Native (0.9%, *n* = 3), Asian (0.9%, *n* = 3), Central/South American or Caribbean (0.6%, *n* = 2), South Asian (0.6%, *n* = 2), Pacific Islander (0.3%, *n* = 1), or a race not listed (0.3%, *n* = 1). Approximately one-quarter (25.6%, *n* = 81) of the participants reported being of Latin American origin or descent.

Participants’ education level was relatively high, with 91.3% (*n* = 289) having some form of college-level education and 52.1% (*n* = 165) having obtained at least their bachelor’s degree from a college or university. Most participants reported receiving annual household income between $35,000 and $49,999 (30.0%, *n* = 95), followed by $50,000 to $74,999 (23.7%, *n* = 75), $20,000 to $34,999 (24.6%, *n* = 78), $75,000 to $99,999 (11.0%, *n* = 35), more than $100,000 (7.3%, *n* = 23), $10,000 to $19,999 (2.5%, *n* = 8), and less than $10,000 (0.9%, *n* = 3). See Table 1 for sociodemographic characteristics and Supplementary Table S1 for childhood adversity and psychosocial outcomes-related characteristics.

### 4.2. Preliminary Analyses

Pairwise Pearson correlations, means, and standard deviations are presented in Table 2. Childhood exposure to threat was positively correlated with emotion regulation difficulties (*r* = 0.68), mental health behaviors (*r* = 0.64), and health risk behaviors (*r* = 0.71). Similarly, reported childhood exposure to deprivation was correlated with emotion regulation difficulties (*r* = 0.57), mindfulness (*r* = −0.62), mental health behaviors (*r* = 0.53), and health risk behaviors (*r* = 0.54). Emotion regulation difficulties were positively associated with both mental health and health risk behaviors (*r* = 0.67 and 0.68, respectively), whereas mindfulness was negatively associated with both symptom types (*r* = −0.63 and −0.47, respectively). VIF for the models ranged from 1.15 to 2.76. Since they fell below the established threshold of 5, multicollinearity was not a substantial concern. Although childhood threat and deprivation were moderately correlated, they did not meaningfully inflate the variance of the regression coefficients. See Supplementary Table S2 for more information on means, standard deviations, and correlations with confidence intervals between CTQ threat and deprivation subscales and individual outcome variables.

#### 4.2.1. Childhood Exposure to Threat and Mental Health Symptoms’ Indirect Effects

Childhood exposure to threat was significantly associated with mental health behaviors (*β* = 1.14, *SE* = 0.20, *p* < 0.001, 95% CI [0.74, 1.53]) and health risk behaviors (*β* = 0.80, *SE* = 0.11, *p* < 0.001, 95% CI [0.59, 1.01]). Childhood threat was not related to current disposition mindfulness (*β* = −0.11, *SE* = 0.059, *p* = 0.059, 95% CI [−0.23, 0.004]). ER difficulties were significantly associated with both mental health behaviors (*β* = 1.33, *SE* = 0.18, *p* < 0.001, 95% CI [0.98, 1.69]) and health risk behaviors (*β* = 0.51, *SE* = 0.096, *p* < 0.001, 95% CI [0.33, 0.71]) symptom scores. Based on 1000 bootstrapped samples of the models (Table 3), childhood threat exposure and mental health behaviors were significantly indirectly related through ER difficulties (*β* = 0.72, *SE* = 0.13, *p* < 0.001, 95% CI [0.50, 1.02]). Similarly, threat and health risk behaviors were significantly indirectly related through ER difficulties (*β* = 0.29, *SE* = 0.064, *p* < 0.001, 95% CI [0.18, 0.43]). See Table 2 and Table 3, and Supplementary Figures S1–S8.

#### 4.2.2. Childhood Exposure to Deprivation and Mental Health Symptoms’ Indirect Effects

Childhood exposure to deprivation was significantly associated with dispositional mindfulness (*β* = −0.51, *SE* = 0.061, *p* < 0.001, 95% CI [−0.63, −0.39]), which in turn was significantly associated with both mental health behaviors (*β* = −1.39, *SE* = 0.17, *p* < 0.001, 95% CI [−1.71, −1.06]) and health risk behaviors (*β* = −0.18, *SE* = 0.091, *p* = 0.049, 95% CI [−0.36, −0.0006]) symptoms. Based on 1000 bootstrapped samples (Table 3), childhood deprivation exposure and mental health behaviors were significantly and indirectly related through dispositional mindfulness (*β* = 0.71, *SE* = 0.13, *p* < 0.001, 95% CI [0.49, 1.01]) and ER difficulties (*β* = 0.25, *SE* = 0.096, *p* = 0.002, 95% CI [0.09, 0.49]). Similarly, childhood deprivation exposure and health risk behaviors were significantly and indirectly related through dispositional mindfulness (β = 0.11, SE = 0.065, *p* = 0.004, 95% CI [0.002, 0.26]) and ER difficulties (*β* = 0.11, *SE* = 0.053, *p* = 0.002, 95% CI [0.04, 0.24]). See Table 2 and Table 3, and Supplementary Figures S1–S8.

## 5. Discussion

The current study examined the impact of two theoretically derived and empirically supported transdiagnostic mechanisms—*ER* and *mindfulness*-–on the relationship between different experiences of childhood maltreatment and symptomology. While cross-sectional in nature, the current study is the first of its kind to examine the unique associations between exposure to childhood threat (i.e., abuse) and deprivation (i.e., neglect), respectively, and mental health behaviors and/or health risk behaviors among young adult SMM. Replicating and extending the larger literature on childhood maltreatment, threat and deprivation were both positively associated with self-reported mental health behaviors (i.e., anxiety, depression, PTSD, and suicidality) and health risk behaviors (i.e., smoking, alcohol use, and sexual compulsivity) symptoms among young adult SMM (i.e., ages 18–35). Grounded in the DMAP framework, the present study went a step further by examining the role of two highly promising transdiagnostic factors—*ER* and *mindfulness*—in the relationship between threat, deprivation, and symptomology among young adult SMM. These relations are particularly important to examine among SMM because they experience disproportionately high rates of psychopathology and suicide; these health inequities are driven, in part, by minority stressors unique to SMM, which can be intertwined with their experiences of childhood maltreatment [113].

It is notable that *both threat* and *deprivation* were associated with *both* mental health and health risk behaviors in this population. This is perhaps unsurprising, given the high prevalence and comorbidity of psychopathology across the mental health–health risk behavior spectra, as well as the high incidence of, and overlap between, various forms of childhood maltreatment experienced by SMM in part due to their minoritized status. It is important to highlight that the relationship between childhood threat and current mental health and health risk behaviors was characterized by large effect sizes (*r*’s = 0.64–0.71), whereas the relationship between deprivation and symptomology was only modest, with moderate effect sizes (*r*’s = 0.53–0.54). The strength of the association between threat and symptoms of psychopathology is consistent with a wealth of prior research in the field of developmental psychopathology, finding that early experiences of violence alter emotional functioning by compromising the development of key emotion-related cortical and subcortical circuits in ways that facilitate the rapid identification of danger in one’s environment [13]. These resulting deficits in emotional processing, learning, reactivity, and regulation—in addition to alterations in attention and memory for emotionally laden stimuli [114,115]—dynamically interact with one’s environment in ways that potentiate the risk for multiple forms of psychopathology, including mental health and health risk behaviors [24,116]. In other words, experiences like child abuse alter brain structure and function in ways that heighten the salience of potentially dangerous or threatening stimuli, which, when combined with ER deficits and other contextual risk factors (e.g., minority stress), may lead to the onset of psychopathology. As many forms of minority stress experiences in the environment are threat-based in nature (e.g., discrimination, peer-bullying, and microaggressions), they may also reinforce the pathway between threat-based childhood trauma (that occurs in one’s household) and psychopathology among SMM and therefore create a heightened relationship.

### 5.1. Threat Models

***ER.*** *ER* difficulties emerged as a significant indirect effect in the relationship between childhood *threat* (i.e., abuse) and both mental health and health risk behaviors symptomology among young adult SMM. More specifically, childhood threat—specifically, physical, emotional, and/or sexual abuse—was associated with lacking access to effective *ER* strategies among SMM. These ER difficulties, in turn, were associated with elevated symptoms of anxiety, depression, PTSD, and suicidality. This is consistent with extant theoretical [117,118] and empirical [118,119] research with predominantly heterosexual samples on the links between childhood abuse, ER difficulties, and psychopathology [83]. Previous studies have demonstrated environmental impacts of chronic threat exposure in childhood on neural development that inhibits cognitive reappraisal and heightens reactivity to emotional cues [41,120]. The implication of these studies is that the decreased processing of the wider context, coupled with the increased effort required to engage in perspective taking, necessitates that SMM may have to exert a higher level of effort to regulate their heightened emotions. It is therefore possible that this higher threshold for emotion regulation may break down more easily under stressful situations [121]. Though the findings for this study are consistent with those of other research studies examining childhood threat’s effects on ER, it is worth noting that these results may have greater import for this population, as SM may be at greater risk of experiencing stressful circumstances and continued chronic threat. As mentioned, Hatzenbuehler’s mediation framework [35] hypothesizes that stigma towards SM increases stress exposure, leading to elevations in risk and problems across domains, including ER, and that these problems in turn serve as an intervening variable in the relationship between stigma-related stress and psychopathology. Young adult SMM experiencing impaired ER from childhood threat may then suffer by a magnitude due to ongoing minority stressors across multiple domains. A large study of youth (*N* = 11,622) found that both interpersonal and structural stressors targeting SM youth contributed to mental health and health risk behaviors [114], with those residing in areas of the U.S. with greater structural stigma endorsing higher rates of psychopathology than their peers. Continual minority stressors experienced at the individual and societal level can then be seen as a type of chronic threat exposure compounding an already taxed ER system [122].

***Mindfulness.*** Due to the lack of prior research in this area, we did not make any specific predictions with respect to threat and mindfulness. The results revealed that *mindfulness* did not emerge as an intervening variable in the relationship between childhood *threat* and present-day mental health behaviors and health risk behaviors among young adult SMM. This null finding was somewhat surprising in light of prior research showing that those who experience threat and/or deprivation early in life (i.e., before the age of 18) demonstrate lower levels of trait mindfulness in adulthood [70]. A prior mindfulness intervention study with sexual minority women also found that those who experienced higher parental emotional and verbal abuse in childhood responded better to the intervention, emphasizing the potential of mindfulness to disrupt the pathway between parental abuse and ER challenges, including emotional eating as a study outcome [123]. However, the vast majority of the limited research in this area has been cross-sectional in nature and either exclusively focused on heterosexual populations or failed to report or collect data on participants’ sexual orientation altogether. They also tend to rely on a unitary “maltreatment” construct, which precludes our ability to examine the potential unique impact of exposure to threat vs. deprivation on mindfulness. In sum, although *ER* and *mindfulness* are related constructs, our results show that *ER* problems in particular represent a promising mechanism through which childhood abuse (i.e., threat) may contribute to mental health and health risk behaviors alike, from symptoms of depression to smoking to sexual compulsivity.

### 5.2. Deprivation Models

***ER*.** The results revealed that the indirect effect between childhood deprivation and both mental health and health risk behaviors was statistically significant. This is consistent with the literature on *ER*, where deprivation alone—i.e., in the absence of other forms of maltreatment—is less likely to be linked to *ER* difficulties compared to threat [119,124]. Theoretical models such as McLaughlin’s transdiagnostic model [19] suggest that threat and deprivation affect psychopathology through different domains, with threat being linked most strongly with *ER* difficulties and deprivation executive function difficulties. Deprivation’s effects on *ER* are hypothesized to work through more indirect mechanisms through the complex interaction with other factors [121]. Studies that have found links between emotion dysregulation and deprivation show that children exposed to deprivation may be prone to *overregulation* of negative emotion as well as a general lack of recognition of their own internal processes [125]. As many of the items on the DERS require a recognition of when an individual is upset and focuses on a lack of inhibition when an individual is experiencing negative emotions, those who ignore and inhibit negative emotions are unlikely to score highly on the measure.

***Mindfulness.*** The results showed that trait *mindfulness* was a significant indirect effect in the relationship between deprivation and both mental health and health risk behaviors. We found that *deprivation* (vs. threat) was uniquely associated with lower levels of trait mindfulness in young adult SMM’s daily lives, which, in turn, was associated with higher endorsement of a range of mental health and health risk behaviors. This compelling finding is consistent with past research showing that childhood deprivation may compromise development in such a profound way and that it may lead to deficits in executive functioning. This is important because the neural architecture underlying these developing executive functioning processes is also fundamental to one’s entire attentional system, including children’s capacity for—and tendency towards—mindful awareness. Executive functioning—encompassing complex processes such as memory, attention, inhibition, and cognitive control—can be thought of as the foundation necessarily influencing—and, in cases of deprivation, potentially *constraining*—the development of children’s eventual “inherent” level of mindfulness as they transition from childhood to adulthood [72,74,75,126]. In a large meta-analysis (N = 31,188), exposure to early-life deprivation was strongly associated with deficits in inhibitory control and working memory [71], both of which are related to dispositional mindfulness. Indeed, although cross-sectional, we found evidence for a significant indirect effect of trait-level *mindfulness* in the relationship between childhood deprivation and adult psychopathology, including mental health and health risk behaviors. This finding supports and extends the emerging research in this area documenting the unique impacts of *deprivation* in childhood on young adult SMM in particular.

### 5.3. Limitations/Future Directions

While promising, the current study has a number of limitations to guide future research on the impact of childhood trauma among young adult SMM, an understudied, yet high-risk, population. A primary limitation of the current study is that it was cross-sectional in nature, testing indirect effects, limiting our ability to prospectively examine mechanisms across time and directionality of constructs. Therefore, future longitudinal studies have the potential to elucidate a richer, more temporally nuanced understanding of the mechanisms driving long-term maladaptive outcomes following early-life adversity and trauma. Another limitation of our study was the potentially differing timeframes across measures that may have introduced increased heterogeneity into our findings and results. Regarding our sample and recruitment strategy, there are limits to the generalizability of our findings. For example, our inclusion criteria required that participants had ever had sex with a man; as such, this may have excluded individuals with a potential trauma history that has impacted their behavior, less sexually active SMM, younger SMM, or individuals who have not yet engaged in sexual activity. Furthermore, sexual orientation comprised sexual identity, sexual behavior, and sexual attraction [127]. In our recruitment strategy, we included men who have sex with men (a behavior) along with sexual identity (gay and bisexual) into one category. Future studies should be more intentional in their examination of sexual orientation. Additionally, future studies should not require sexual activity for participation unless relevant to study outcomes.

Furthermore, another important limitation is the composition of the sample, as 55.38% identified as white and all of whom identified as cisgender. In our study, we dichotomized race into People of Color and White in efforts to preserve power for our analyses for reliable results. Yet, it is well-documented that People of Color may experience increased rates of mental health concerns, early childhood adversities, and minority stressors due to the impacts of racism [128]. As such, future studies should replicate our findings with predominantly communities of color. Future studies should also include other sexual and gender minority populations, especially trans men and boys. Future work with larger samples may want to carefully and intentionally assess and incorporate participants’ intersecting identities and examine how different types of minority stressors may interact with non-identity-based forms of *threat* and *deprivation* to confer risk for—or protect against—the development of psychopathology among sexual minorities. Furthermore, future work could consider examining the unique or relative influence of specific types of threat (e.g., sexual abuse) and *deprivation* (e.g., emotional neglect) trajectories of mental health and health risk behaviors among SMM across development. Given the potentially divergent developmental trajectories stemming from experiences of threat vs. those of *deprivation*, future studies should strive for comprehensiveness and specificity in their assessment of early interpersonal trauma. Depending on the context, future researchers should also consider assessing frequency, severity, and duration, in addition to the identity/relationship of the perpetrator(s) of the child; this would allow for a more nuanced, finer-grained understanding of the long-term impact of these experiences among SMM, ideally informing trauma-informed prevention and intervention efforts. The utilization of a composite score also prevents us from determining outcome-specific conclusions that could further inform targeted interventions. Additionally, the utilization of varying timeframes for the assessment of various measures may introduce heterogeneity into the findings. Finally, future studies should consider examining the influence of other variables that intersect with adversity pathways such as internalized stigma and social support.

## 6. Clinical Implications and Conclusions

The study findings have several clinical implications. First, the findings may inform suicide prevention efforts and broader mental health initiatives such as psychoeducation within the context of PTSD, anxiety, and depression and their associations with childhood emotional neglect and deprivation. For example, one prior study found that childhood emotional neglect is associated with greater odds of suicide attempts [129]. Second, given the increased prevalence rates of childhood maltreatment among SMM, as well as the continued lifetime minority stressors from all spheres of influence, the use of mindfulness and acceptance-based treatments specifically tailored to SMM experiences is a promising clinical direction in decreasing symptomology [130]. The ability to act in a conscious, non-judgmental manner, as well as recognition of internal processes, acts as a buffer for various associations of SM identity discrimination on mood lability–particularly as it relates to SI [131]. One adapted mindfulness-based intervention that embeds minority stress theory into its protocol for SMM has shown success in mitigating the cumulative experiences of these stressors via mindfulness, as well as increasing attention to internal processes and engagement with therapeutic services [123]. Furthermore, there is a robust and growing body of literature underscoring the importance of culturally adapting evidence-based interventions for sexual and gender minorities in addition to communities of color, given the unique intersection of trauma histories, societal stigma, and culture [132,133,134,135,136]. In conclusion, our study underscores the complexity of two promising mechanisms (i.e., *ER* and *mindfulness*) in the relations between childhood threat and deprivation and their associations with mental health and health risk behaviors among SMM.

## Figures and Tables

**Table 1:** Sociodemographic characteristics of participants (N = 317).

Sample Characteristics	%	*n*
Race/Ethnicity		
American Native or Alaska Native	0.9	3
Asian	0.9	3
South Asian	0.6	2
Black or African American	35.6	113
Pacific Islander	0.3	1
White	59.3	188
Multiple	1.3	4
Middle Eastern	0	0
Central/South American or Caribbean	0.6	2
Other	0.3	1
Hispanic/Latinx		
Yes	25.6	81
No	74.4	236
Sexual Orientation		
Bisexual	18	57
Gay	80.8	256
Pansexual	1.3	4
Not listed here	0	0
Not sure	0	0
Household Income		
Less than $10,000	0.9	3
$10,000 to $19,000	2.5	8
$20,000 to $34,999	24.6	78
$35,000 to $49,999	30	95
$50,000 to $74,999	23.7	75
$75,000 to $99,999	11	35
More than $100,000	7.3	23
Education		
Less than high school	0	0
High school or GED only	8.8	28
Some college, no degree	31.9	101
Bachelor’s degree	52.1	165
Graduate degree	7.3	23

**Table 2:** Means, Standard Deviations, and Intercorrelations among Study Variables (*n* = 317).

Variables	1	2	3	4	5	6
1. CTQ Threat	-					
2. CTQ Deprivation	0.68 ** [0.61, 0.73]	-				
3. Emotion dysregulation	0.68 ** [0.61, 0.73]	0.57 ** [0.49, 0.64]	-			
4. Mindfulness	−0.48 ** [−0.56, −0.39]	−0.62 ** [−0.69, −0.55]	−0.59 ** [−0.65, −0.51]	-		
5. Mental Health Behaviors	0.64 ** [0.57, 0.70]	0.53 ** [0.45, 0.61]	0.67 ** [0.60, 0.73]	−0.63 ** [−0.69, −0.56]	-	
6. Health Risk Behaviors	0.71 ** [0.64, 0.77]	0.54 ** [0.44, 0.62]	0.68 ** [0.61, 0.75]	−0.47 ** [−0.56, −0.36]	0.72 ** [0.66, 0.78]	-
M	34.90	25.98	46.47	3.66	−0.00	0.55
SD	12.63	7.67	11.11	0.90	3.44	1.69

M and SD are used to represent mean and standard deviation, respectively. *p* < 0.01 **. Mental health behavior is a composite sum of z-scored PHQ-9, GAD-7, PCL-5, and SBQ-R. Health risk behavior is a composite sum of z-scored SCS-2 and AUDIT plus lifetime smoking history (binary). Means near zero are indicative of z-score centering.

**Table 3:** Direct and Indirect Effect Estimates of Bootstrapped Mediation Analyses.

	Direct Effect Estimates				Indirect Effect Estimates			
	Estimate			95% CI	Bootstrap Estimate			95% CI Bootstrap Bias Corrected
Paths	*β*	*SE*	*p*		*β*	*SE*	*p*	
Outcome: Mental Health Behaviors								
Threat → Emotion Dysregulation → Mental Health Behaviors	1.14	0.20	<0.001 *	(0.71, 1.58)	0.72	0.13	<0.001 ^*	(0.50, 1.02)
Deprivation → Emotion Dysregulation → Mental Health Behaviors	0.18	0.19	0.34	(−0.17, 0.52)	0.25	0.096	0.002 ^*	(0.09, 0.49)
Threat → Mindfulness → Mental Health Behaviors	1.71	0.17	<0.001 *	(1.38, 2.07)	0.16	0.09	0.074	(−0.01, 0.34)
Deprivation → Mindfulness → Mental Health Behaviors	−0.29	0.20	0.15	(−0.70, 0.09)	0.71	0.13	<0.001 ^*	(0.49, 1.01)
Outcome: Health Risk Behaviors								
Threat → Emotion Dysregulation → Health Risk Behaviors	0.80	0.11	<0.001 *	(0.61, 1.04)	0.29	0.064	<0.001 ^*	(0.18, 0.43)
Deprivation→ Emotion Dysregulation → Health Risk Behaviors	−0.07	0.10	0.44	(−0.31, 0.11)	0.11	0.053	0.01 *	(0.03, 0.24)
Threat → Mindfulness → Health Risk Behaviors	1.08	0.10	<0.001 *	(0.88, 1.27)	0.016	0.016	0.27	(−0.004, 0.06)
Deprivation → Mindfulness → Health Risk Behaviors	−0.08	0.12	0.53	(−0.35, 0.15)	0.11	0.065	0.04 *	(0.002, 0.26)

*β* = Standardized effect estimate. Direct effects are computed from OLS regression and indirect effects are estimated using nonparametric bootstrapping (at 1000 samples) with bias-adjusted confidence intervals. ^ indicates that it survived Bonferroni correction and * indicates statistical significance (*p*-value threshold < 0.05). All models were controlled for age, race, education, and the countermeasure childhood maltreatment variable.

## Data Availability

De-identified data can be accessed at https://osf.io/xydjk/ (accessed on 1 March 2026).

## Supplementary Materials

The following supporting information can be downloaded at: https://www.mdpi.com/article/10.3390/ijerph23050609/s1, Figure S1. Indirect pathway from Threat to Mental Health Behaviors via Emotion, Figure S2. Indirect pathway from Deprivation to Mental Health Behaviors via Emotion Dysregulation, Figure S3. Indirect pathway from Threat to Mental Health Behaviors via Mindfulness, Figure S4. Indirect pathway from Deprivation to Mental Health Behaviors via Mindfulness, Figure S5. Indirect pathway from Threat to Health Risk Behaviors via Emotion Regulation, Figure S6. Indirect pathway from Deprivation to Health Risk Behaviors via Emotion Regulation, Figure S7. Indirect pathway from Threat to Health Risk Behaviors via Mindfulness, Figure S8. Indirect pathway from Deprivation to Health Risk Behaviors via Mindfulness; Table S1: Sample Characteristics by Childhood Adversity and Psychosocial Outcomes (N = 317); Table S2: Means, standard deviations, and correlations with confidence intervals between CTQ threat and deprivation subscales and individual outcome variables.
