# Supplementary material for: Childhood Threat and Deprivation and Links to Mental Health Behaviors and Health Risk Behaviors Among Young Sexual Minority Men: The Differential Roles of Mindfulness and Emotion Regulation
Source: Int J Environ Res Public Health. 2026 May 5;23(5):609. doi: 10.3390/ijerph23050609 (PMC13206307; doi:10.3390/ijerph23050609)
Supplement: Supplementary file 1 [file ijerph-23-00609-s001.zip › ijerph-3967529-supplementary.pdf]

Supplemental Material

Supplemental Figures S1–S8: Indirect Effect Estimates of Bootstrapped Mediation Analyses

Figure S1. Indirect pathway from Threat to Mental Health Behaviors via Emotion

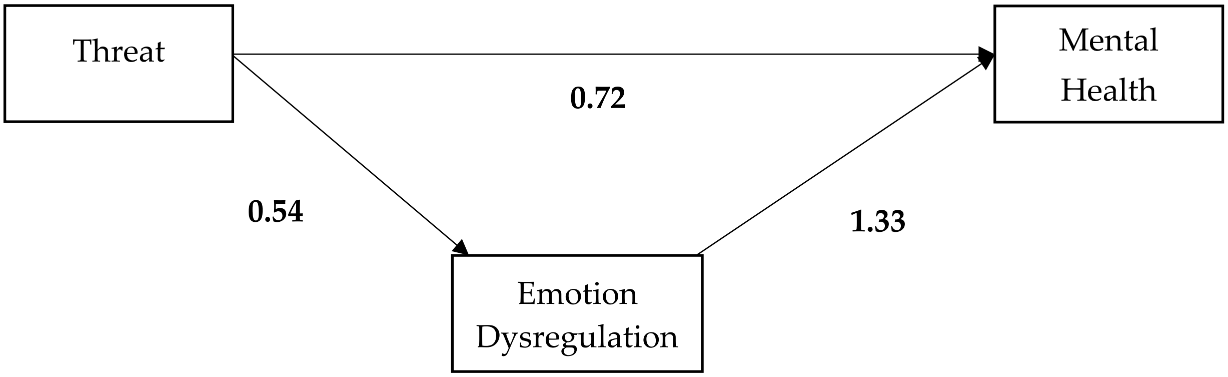

Figure S2. Indirect pathway from Deprivation to Mental Health Behaviors via Emotion

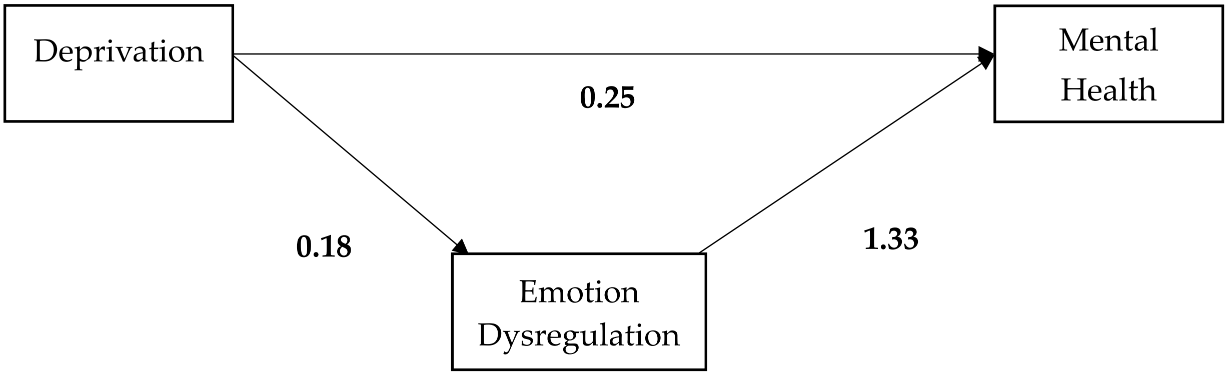

Figure S3. Indirect pathway from Threat to Mental Health Behaviors via Mindfulness

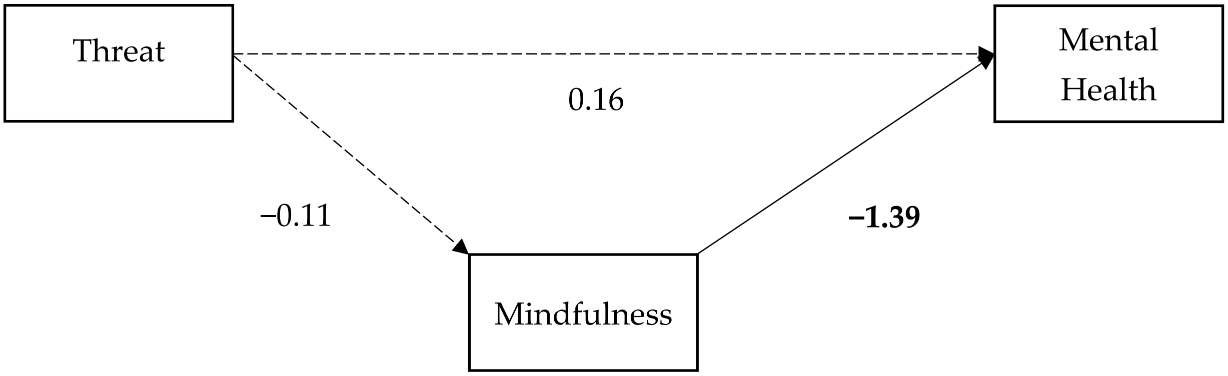

Figure S4. Indirect pathway from Deprivation to Mental Health Behaviors via Mindfulness

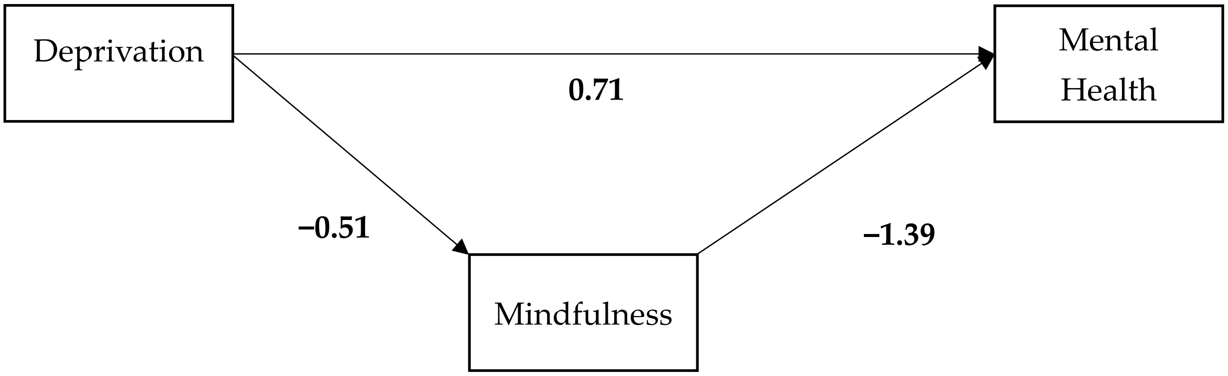

Figure S5. Indirect pathway from Threat to Health Risk Behaviors via Emotion Regulation

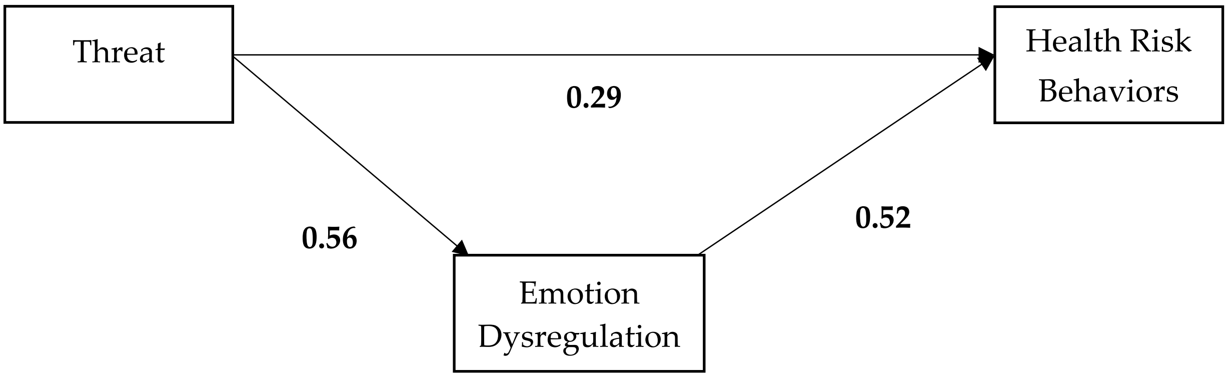

Figure S6. Indirect pathway from Deprivation to Health Risk Behaviors via Emotion Regulation

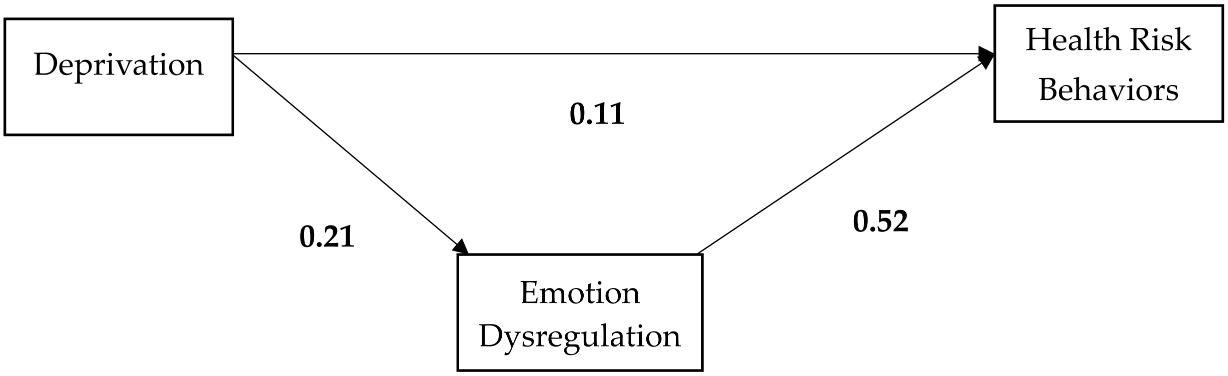

Figure S7. Indirect pathway from Threat to Health Risk Behaviors via Mindfulness

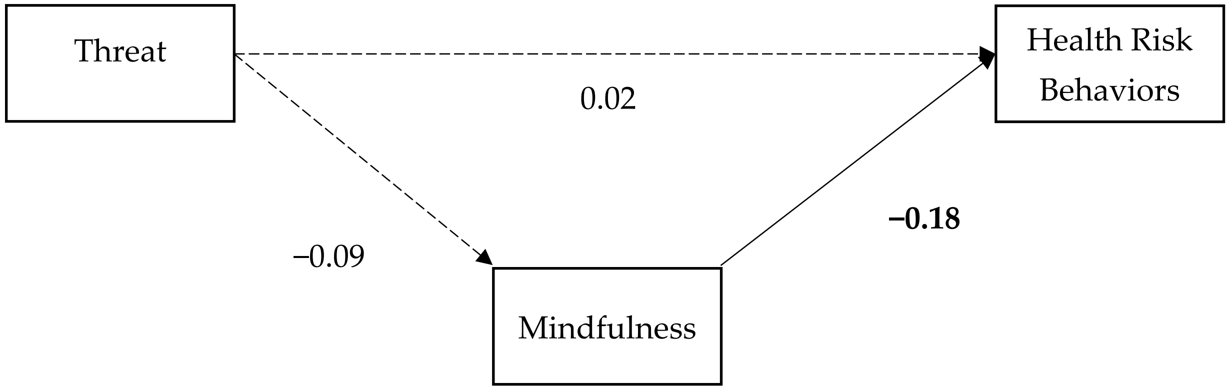

Figure S8. Indirect pathway from Deprivation to Health Risk Behaviors via Mindfulness

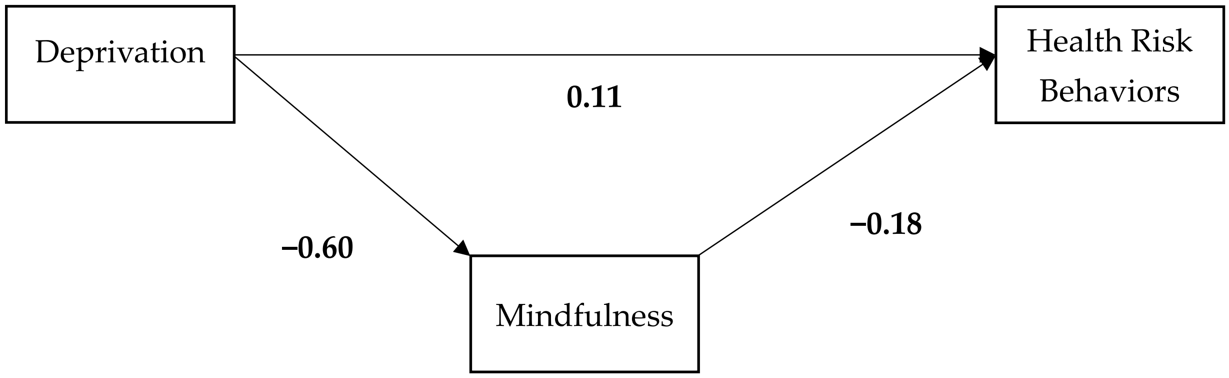

Note: Dotted lines represent non-significant paths.

Supplemental Table S1: Sample Characteristics by Childhood Adversity and Psychosocial Outcomes (*n*=317)

**Characteristics**	**n (%)**
**Emotional Abuse**	
None/Minimal	80 (25.24)
Low to Moderate	83 (26.18)
Moderate to Severe	91 (28.71)
Severe to Extreme	63 (19.87)
**Physical Abuse**	
None/Minimal	82 (25.87)
Low to Moderate	14 (4.42)
Moderate to Severe	65 (20.50)
Severe to Extreme	156 (49.21)
**Sexual Abuse**	
None/Minimal	70 (22.08)
Low to Moderate	17 (5.36)
Moderate to Severe	68 (21.45)
Severe to Extreme	162 (51.10)
**Emotional Neglect**	
None/Minimal	67 (21.14)
Low to Moderate	84 (26.50)
Moderate to Severe	98 (30.91)
Severe to Extreme	68 (21.45)
**Physical Neglect**	
None/Minimal	73 (23.30)
Low to Moderate	13 (4.10)
Moderate to Severe	45 (14.20)
Severe to Extreme	186 (58.68)
**Depression Severity (PHQ-9)**	
None/Minimal	77 (24.29)
Mild	66 (20.82)
Moderate	116 (36.59)
Moderately Severe	56 (17.67)
Severe	2 (0.63)
**Anxiety Severity (GAD-7)**	
Minimal	97 (30.60)
Mild	116 (36.59)
Moderate	96 (30.28)
Severe	8 (2.52)
**PTSD Symptom Severity (PCL-C)**	
Below Clinical Threshold	141 (44.48)

Probable PTSD	176 (55.52)
**Suicide Risk (SBQ-R)**	
Not at Risk	177 (55.84)
At Risk	140 (44.16)
**Sexual Compulsivity (SCS)**	
Low Sexual Compulsivity	239 (78.62)
High Sexual Compulsivity	65 (21.38)
**Hazardous Alcohol Use (AUDIT-C)**	
Non-hazardous	78 (32.5)
Hazardous	162 (67.5)
**Lifetime Cigarette Smoking**	
Never Smoked	134 (42.27)
Ever Smoked	183 (57.72)
**Emotional Dysregulation (DERS)**	
Low Dysregulation	149 (47.00)
High Dysregulation	168 (53.00)
**Dispositional Mindfulness (MAAS)**	
Low Mindfulness	98 (30.91)
Moderate Mindfulness	108 (34.07)
High Mindfulness	111 (35.02)

Supplemental Table S2: Means, standard deviations, and correlations with confidence intervals between CTQ threat and deprivation subscales and individual outcome variables

Variable	*M*	*SD*	1	2	3	4	5	6	7	8
1. CTQ Threat	34.90	12.63								
2. CTQ Deprivation	25.98	7.67	0.68** [0.61, 0.73]							
3. Depression (PHQ-9)	9.16	5.50	0.64** [0.57, 0.70]	0.53** [0.45, 0.61]						
4. Anxiety (GAD-7)	6.99	4.48	0.50** [0.41, 0.57]	0.37** [0.27, 0.46]	0.83** [0.80, 0.87]					
5. PTSD (PCL-5)	41.72	12.95	0.75** [0.70, 0.80]	0.59** [0.51, 0.66]	0.79** [0.75, 0.83]	0.70** [0.64, 0.75]				
6. Suicidality (SBQ-R)	6.54	3.55	0.32** [0.22, 0.42]	0.33** [0.23, 0.43]	0.54** [.45, .61]	0.59** [0.51, 0.66]	0.48** [0.39, 0.56]			
7. Sexual Compulsivity (SCS)	11.73	6.28	0.75** [0.69, 0.79]	0.62** [0.55, 0.69]	0.68** [0.61, 0.74]	0.57** [0.48, 0.64]	0.71** [0.64, 0.76]	0.39** [0.29, 0.48]		
8. Alcohol Use (AUDIT-C)	4.50	1.93	0.33** [0.21, 0.44]	0.13* [0.01, 0.26]	0.31** [0.19, 0.42]	0.28** [0.16, 0.39]	0.38** [0.26, 0.48]	0.35** [0.23, 0.45]	0.19** [0.06, 0.31]	
9. Lifetime Smoking	0.58	0.49	0.06 [−0.05, 0.17]	0.11 [0.00, 0.22]	0.01 [−0.10, 0.12]	−0.02 [−0.13, 0.09]	0.04 [−0.07, 0.15]	0.09 [−0.02, 0.19]	−0.01 [−0.13, 0.10]	0.16* [0.03, 0.28]

Note. M and *SD* are used to represent mean and standard deviation, respectively. Values in square brackets indicate the 95% confidence interval for each correlation. The confidence interval is a plausible range of population correlations that could have caused the sample correlation (Cumming, 2014).
* indicates $p < 0.05$. ** indicates $p < 0.01$.
